# Editorial: Artificial intelligence and anti-cancer drugs: advancing discovery, personalization, and precision oncology

**DOI:** 10.3389/fonc.2026.1988629

**Published:** 2026-09-30

**Authors:** Mohamed El-Shazly, Neeraj Kumar, Sumit Sheoran

**Affiliations:** 1 Pharmacognosy Department, Faculty of Pharmacy, Ain Shams University, Cairo, Egypt; 2 Graduate Institute of Natural Products, College of Pharmacy, Kaohsiung Medical University, Kaohsiung, Taiwan; 3 Feinberg School of Medicine, Northwestern University, Chicago, IL, United States; 4 Babasaheb Bhimrao Ambedkar University, Lucknow, India

**Keywords:** anti-cancer, artificial intelligence, drug discovery, oncology, precision medicine

## Setting the scope: what AI can and cannot yet do

It is fitting that the Topic opens with a broad review of the field itself. Nagrami et al. map AI’s footprint across the entire anti-cancer drug pipeline — target identification, virtual screening, molecular docking, multi-omic integration, and the still-unresolved question of clinical validation. Their review is a useful corrective to hype: AI has undeniably compressed timelines in early discovery, but the harder problems — model interpretability, data quality, and regulatory acceptance — remain largely where they were five years ago. That tension between computational promise and clinical caution runs through nearly every paper in this collection.

## From population averages to the individual patient

Precision oncology has long promised to treat the patient in front of you rather than the “average” patient in a trial. Pradosh et al. take this promise seriously in the context of dosing itself, reviewing model-informed precision dosing and pharmacometrics-based clinical decision support systems across agents such as busulfan, high-dose methotrexate, carboplatin, and several tyrosine kinase inhibitors. Their focus on Asian healthcare settings is a welcome corrective to a literature dominated by Western data: fragmented electronic records, limited assay infrastructure, and unclear regulatory pathways are not footnotes — they are the actual barriers standing between a validated dosing model and a patient who benefits from it.

Molecular stratification is the other half of personalization, and Zhao et al. offer a rigorous demonstration of what that can look like in glioblastoma. Using non-negative matrix factorization on endoplasmic reticulum stress–related DNA methylation profiles, they identify four molecular subtypes, one of which — Subtype 2 — carries a markedly worse prognosis and a distinct immune microenvironment. Their random forest classifier, validated externally on TCGA data with over 92% accuracy, and their exploratory identification of MEK inhibitors as candidate compounds for this high-risk subtype, illustrate how machine learning can turn a static diagnosis into an actionable hypothesis — while the authors themselves are careful to flag that computational signal is not yet biological proof.

## Predicting who will respond — and to what

A recurring ambition across this Topic is prediction: will this patient respond to this regimen? Yan et al. tackle this question at the level of the whole field, systematically reviewing 30 studies and over 14,000 esophageal cancer patients to compare classical machine learning, ensemble methods, and deep learning for predicting treatment response. Their headline finding — that multimodal models outperform single-modality ones, and that deep learning edges out simpler approaches once systems have enough data — is less interesting than their diagnosis of what is holding the field back: opaque methods, poorly justified predictor selection, and a near-total absence of prospective validation.

That same caution — used constructively — animates Babic et al., who take arguably the most direct test of clinical AI utility in this Topic: pitting four large language models against 151 real-world melanoma multidisciplinary tumor board decisions. The best-performing model, ChatGPT-5 Thinking, achieved encouraging concordance, particularly in more standardized, surgery-driven cases. But the more clinically important finding sits in the details — performance gaps between models widened precisely when decisions grew genuinely complex, involving adjuvant or systemic therapy choices.

## Closing the loop: dosing, delivery, and drug safety in the real world

Precision medicine is not only about choosing the right drug — it is about managing it once it is in the patient. Shen et al. contribute a detailed case report of response-adapted neoadjuvant alectinib in ALK-rearranged lung cancer, using serial imaging rather than a fixed treatment duration to time surgery — an elegant, if single-patient, illustration of what individualized treatment scheduling can achieve when clinicians are willing to let response data, rather than protocol convention, drive decisions. Wang et al. turn to a much harder clinical problem — leptomeningeal metastasis from non-small-cell lung cancer — systematically reviewing intrathecal chemotherapy across twelve studies and 544 patients, and candidly concluding that despite genuine clinical benefit, the field still lacks the prospective, standardized evidence needed to define an optimal regimen.

Not every advance in this Topic comes from a purely computational direction. Li et al. use network pharmacology to reverse-engineer the mechanism of a century-old traditional Chinese medicine formula, Liu Wei, showing in a glioma mouse model that its constituents baicalin and luteolin suppress EGFR/PI3K/AKT signaling and enhance CD8^+^ T-cell infiltration — with corroborating clinical case evidence. It is a reminder that “AI in oncology” need not mean deep learning alone; computational network analysis is equally at home unpacking the multi-target logic of a herbal formula as it is screening a virtual chemical library.

Finally, Fan et al. close the loop by looking downstream of approval altogether, mining over 115,000 FAERS pharmacovigilance reports with machine learning to distinguish the adverse-event signatures of the three major CDK4/6 inhibitors in breast cancer — palbociclib’s hematologic burden, abemaciclib’s gastrointestinal and thrombotic signal, ribociclib’s QT and hepatobiliary profile — and grounding these signals against a real institutional cohort.

## Where this leaves us

Taken together, these nine papers resist a simple, triumphant narrative, and that is precisely their value. AI is genuinely accelerating target discovery and molecular stratification; it is beginning to earn a supportive role at the tumor board table; and it is sharpening how we monitor drugs once they reach patients. But almost every contributor to this Topic pauses, in their own way, to say the same thing: retrospective performance is not clinical validation, computational signal is not mechanism, and concordance with expert decisions is not evidence of improved outcomes. We are grateful to all the authors and reviewers who contributed to this Topic, and we hope it serves as both a snapshot of where AI-driven anti-cancer drug research stands today and a candid map of the work still ahead.

